# ‘I do hope more people can benefit from it.’: The qualitative experience of individuals living with osteoarthritis who participated in the GLA:D™ program in Alberta, Canada

**DOI:** 10.1371/journal.pone.0298618

**Published:** 2024-02-21

**Authors:** Ania Kania-Richmond, Lauren A. Beaupre, Geneviève Jessiman-Perreault, Danika Tribo, Jason Martyn, David A. Hart, Jill Robert, Mel Slomp, C. Allyson Jones

**Affiliations:** 1 Bone and Joint Health Strategic Clinical Network, Alberta Health Services, Calgary, Alberta, Canada; 2 Department of Community Health Sciences, Cumming School of Medicine, University of Calgary, Calgary, Alberta, Canada; 3 Department of Physical Therapy, Faculty of Rehabilitation Medicine, University of Alberta, Edmonton, Alberta, Canada; 4 Dalla Lana School of Public Health, University of Toronto, Toronto, Ontario, Canada; 5 Rockyview General Hospital, Alberta Health Services, Calgary, Alberta, Canada; 6 Department of Surgery, Cumming School of Medicine, University of Calgary, Calgary, Alberta, Canada; Örebro University Faculty of Medicine and Health: Orebro universitet Fakulteten for medicin och halsa, SWEDEN

## Abstract

**Introduction:**

The Good Life with osteoArthritis: Denmark (GLA:D^TM^) is an evidence-based program designed for individuals with symptomatic hip and knee osteoarthritis (OA). This program has reported improvement in pain, quality of life and self-efficacy, as well as delays in joint replacement surgery for adults with moderate to severe hip or knee OA. Evaluations of GLA:D^TM^ implementation in several countries have focused on effectiveness, training, and feasibility of the program primarily from the provider perspective. Our objective was to examine how the GLA:D^TM^ program was perceived and experienced by individuals with hip and knee OA to inform on-going program refinement and implementation.

**Methods:**

Thirty semi-structured telephone interviews were conducted with participants who completed the GLA:D^TM^ program in Alberta. An interpretive description approach was used to frame the study and thematic analysis was used to code the data and identify emergent themes and sub-themes associated with participants’ experience and perception of the GLA:D^TM^ program.

**Results:**

Most participants had a positive experience of the GLA:D^TM^ program and particularly enjoyed the group format, although some participants felt the group format prevented one-on-one support from providers. Three emergent themes related to acceptability were identified: accessible, adaptable, and supportive. Participants found the program to be accessible in terms of location, cost, and scheduling. They also felt the program was adaptable and allowed for individual attention and translatability into other settings. Finally, most participants found the group format to be motivating and fostered connections between participants.

**Conclusion:**

The GLA:D^TM^ program was perceived as acceptable by most participants, yet the group format may not be useful for all individuals living with OA. Recommended improvements included adapting screening to identify those suited for the group format, providing program access earlier in the disease progression trajectory, modifying educational content based on participants’ knowledge of OA and finally, providing refresher sessions after program completion.

## Introduction

Osteoarthritis (OA) is a chronic degenerative condition affecting roughly 1 in 8 adult Canadians [1], with an estimated 30% of OA involving the knee, 13% in hips alone and 30% involving both hips and knees [2]. OA is a painful condition and negatively impacts the physical functioning, mental health, and overall health of those with the condition [3, 4]. By 2040, the number of adult Canadians affected by OA is projected to rise to 1 in 4 in part due to the aging population and increases obesity rates [1, 5, 6]. Evidence-based guidelines recommend education, exercise, and weight management as first-line non-operative treatments for hip and knee OA [7]. Yet evidence from an international study conducted in 5 countries indicate that education and exercise were only recommended to patients with hip and knee OA 55% and 67% of the time, respectively [8]. A recent study conducted in Alberta, Canada, found that only 60% of patients scheduled to received total knee replacement reported receiving recommendations for non-surgical treatments [9]. Therefore, many people living with OA do not receive effective management strategies and seeking out advanced levels of care from specialists [10]. The Good Life with osteoArthritis: Denmark (GLA:D^TM^) is one intervention that has the potential to impact these efforts; however our understanding of how participants view their experience with the GLA:D^TM^ program are absent in published literature.

GLA:D^TM^ is an evidence-based program designed for individuals with symptomatic hip and knee OA. It consists of 12 supervised neuromuscular group exercise classes (1-hour classes, 2 times per week over a 6-week period) and 2 structured education sessions (2 classes approximately 60 minutes each) [11]. Several studies have shown substantial pain relief with a reduction of medications, improvement in quality of life and self-efficacy, delay in joint replacement surgery, and a high satisfaction rate for adults with moderate to severe hip or knee OA after completion of the GLA:D^TM^ program [12–15].

Since its launch in Denmark, the GLA:D^TM^ program has been implemented in Canada and several other countries including Australia, China, Switzerland, Austria, and New Zealand [16]. Evaluations of GLA:D^TM^ implementation and feasibility in Ontario, Canada [14], Denmark [11], and Australia [13] have focused on effectiveness of the program and training, program fidelity, adoption, reach, implementation processes, sustainability and based on the perspectives of health care professionals and health administrators. Yet, the impact and success of the implementation processes of GLA:D^TM^ is dependent on whether individuals living with OA perceive the program to be acceptable. Acceptability of an intervention which is considered the extent to which healthcare service is respectful and responsive to user needs, preferences, expectations [17] from the patient perspective is a necessary component of effectiveness [18]. To date, there has been minimal attention in examining, qualitatively, the patient experience of the GLA:D™ program. Two notable studies have been conducted qualitatively evaluating the GLA:D^TM^ program in Australia. Ezzat et al. [19] examined the acceptability of GLA:D^TM^ program delivered via telehealth in Australia and found that participants perceived the program to be acceptable and reported similar benefits, regardless of delivery method [19]. Wallis et al. [20] focused on the barriers and enablers of participation in the GLA:D^TM^ program from a sample of participants–patients and providers—at a large private hospital [20]. Their findings indicate key issues associated with program uptake include program cost, program promotion, and misinformation regarding OA and exercise therapy, resulting in compromised utilization of programs such as GLA:D^TM^. A better understanding of the experiences of those using GLA:D^TM^ will enable on-going adaptation and quality improvement of the program to better address the needs and preferences of individuals living with OA. Our study objective was to qualitatively examine how participation in the GLA:D^TM^ program was perceived and experienced by individuals with hip and knee OA.

## Methods

### Study design

We conducted a qualitative inquiry applying Thorne et al.’s interpretive description approach [21], which is a flexible non-categorical method to qualitative inquiry that aims to generate understanding relevant to clinical practice, to frame the study. This study is part of the broader Alberta GLA:D^TM^ feasibility evaluation, informed by the experiences of patient advisors who are engaged with the Bone and Joint Health Strategic Clinical Network in Alberta through their participation in network meetings and working groups. The overall evaluation [22] used a staged mixed methods approach, conceptually informed by the RE-AIM framework [23]. Reporting is in accordance with the Consolidated Criteria for Reporting Qualitative Research (COREQ) checklist (see S1 Table for completed checklist) [24]. Ethics approval for this study was received from the Health Research Ethics Review Board of the University of Alberta (Pro00068308).

### Study setting

This evaluation was conducted in Canada’s fourth largest province, Alberta, which has a population of approximately 4.5 million residents spread across five health regions [25]. Approximately 50% of the population resides in two metropolitan centers: Calgary and Edmonton. Alberta has a single-payer health care system available to all Albertans; however, residents also have access to both publicly funded and private rehabilitation services [26].

### Participant selection

A purposive sample of individuals who completed the GLA:D^TM^ program was generated using a maximum variation sampling strategy to maximize diversity of the participants across geography (i.e., rural and urban locations), clinical settings (i.e., public and private payment models), and gender, with the intent to identify common patterns across the diverse contexts of program delivery. Participants were included if they met three criteria: 1) adults (≥18 years of age), 2) living with symptomatic hip or knee OA, and 3) attended the GLA:D^TM^ program between January 2017 to December 2018 in Alberta. Participants were recruited from 9 clinics of the initial cohort of 12 clinics that implemented the GLA:D^TM^ program. This original cohort of clinics that took part in the program were both public (e.g., public healthcare centres, hospitals) (n = 7) and private clinical settings (e.g., multi-disciplinary rehabilitation clinics, physiotherapy clinics) (n = 5). These sites were located across Alberta, in rural (i.e., population less than 25,000), urban (i.e., population of 25,000 to < 500,000) and metropolitan centres (i.e., population >500,000) [27].

Recruitment took place during the educational component of the program. Program participants were informed about the GLA:D^TM^ feasibility evaluation and completed a ‘consent to be contacted’ form if they were interested in participating. A research study team member (AKR) contacted interested participants, ensured that they met the inclusion criteria and, if so, booked a telephone interview.

### Data collection

Thirty semi-structured one-on-one telephone interviews were conducted by the research team (AKR, EM) with participants within, on average, four months after completing the GLA:D^TM^ program. Prior to commencing an interviewing, study consent was reviewed by interviewing researcher with the participant. Participants were asked to verbally indicate their consent for participation, which was then recorded by the researcher as part of the interview record. A semi-structured interview guide was used, which was revised to include three additional questions based on a preliminary review of the initial set of interviews (S1 File). The guide consisted of questions that explored how participants learned about GLA:D^TM^, what motivated them to take the program, how they experienced the different program components (i.e., the education sessions, the exercise sessions), how the program could be improved, the program benefits, and whether they valued the program. Data collection was completed when data saturation was determined, which is the point when subsequent interviews do not generate new insights [28].

### Data analysis

All interviews were audio recorded, de-identified, and transcribed verbatim for analytic purposes. NVIVO Pro12 software was used to support data management and the analytic process. Transcripts and analysis were not returned to participants for comment or feedback. A thematic analysis approach [29] was used to develop descriptive codes and categories. The analysis was conducted by two researchers (DT and AKR). Initial data coding was compared between researchers to establish agreement on descriptive code categories and data interpretation. Descriptive analysis was followed by an interpretive analysis, whereby, through several cycles, the descriptive categories were clustered and re-clustered to inductively identify the emergent themes and sub-themes. Emergent themes were then cross-referenced with the data and descriptive codes to ensure full coverage of participants’ experiences and perceptions of the GLA:D^TM^ program. This analytic output was reviewed by two additional research team members (LAB and CAJ) to ensure logical organization of the clustered categories and alignment with the study objectives. This led to further refinement of the sub-themes and major themes to minimize overlap and redundancy. To enhance the quality of the analytic output, bracketing and code-recode strategy were used. Bracketing in the form of analytic memos was used by the coders during the analytic process [30]. Meetings to discuss the analysis also included discussions of personal reflections that enabled team members to unpack their presumptions and perspectives in relation to emergent findings. For the code-recode strategy, coders undertook repetitive analyses of data segments, comparing their own coding for consistency, and further refinement of emergent categories.

### Research team and reflexivity

Recognizing the potential impact of a researcher’s knowledge and experience to influence the analytic process, the research teams’ backgrounds are described here. Interviews were conducted by two experienced non-clinician qualitative researchers (AKR, EM). AKR holds a PhD and EM holds a MSc, both in health services research. Data analysis was conducted by AKR and DT, who completed a Master’s in Nursing during the time of this study and did not have prior knowledge of GLA:D^TM^. Analytic reviews were held with the two evaluation co-leads (LAB and CAJ), who are both senior academic researchers in bone and joint health, and experienced physical therapists; CAJ is in active clinical practice and delivers training of the GLA:D^TM^ program. All researchers involved in the data collection and analysis identified as female. The research team did not have any interactions with the study participants prior to the interviews and were not affiliated with any participating sites delivering the GLA:D^TM^ program. Interviewing researchers introduced themselves to participants by name and described their role in relation to the study.

## Results

### Participant description

**Fig 1:**
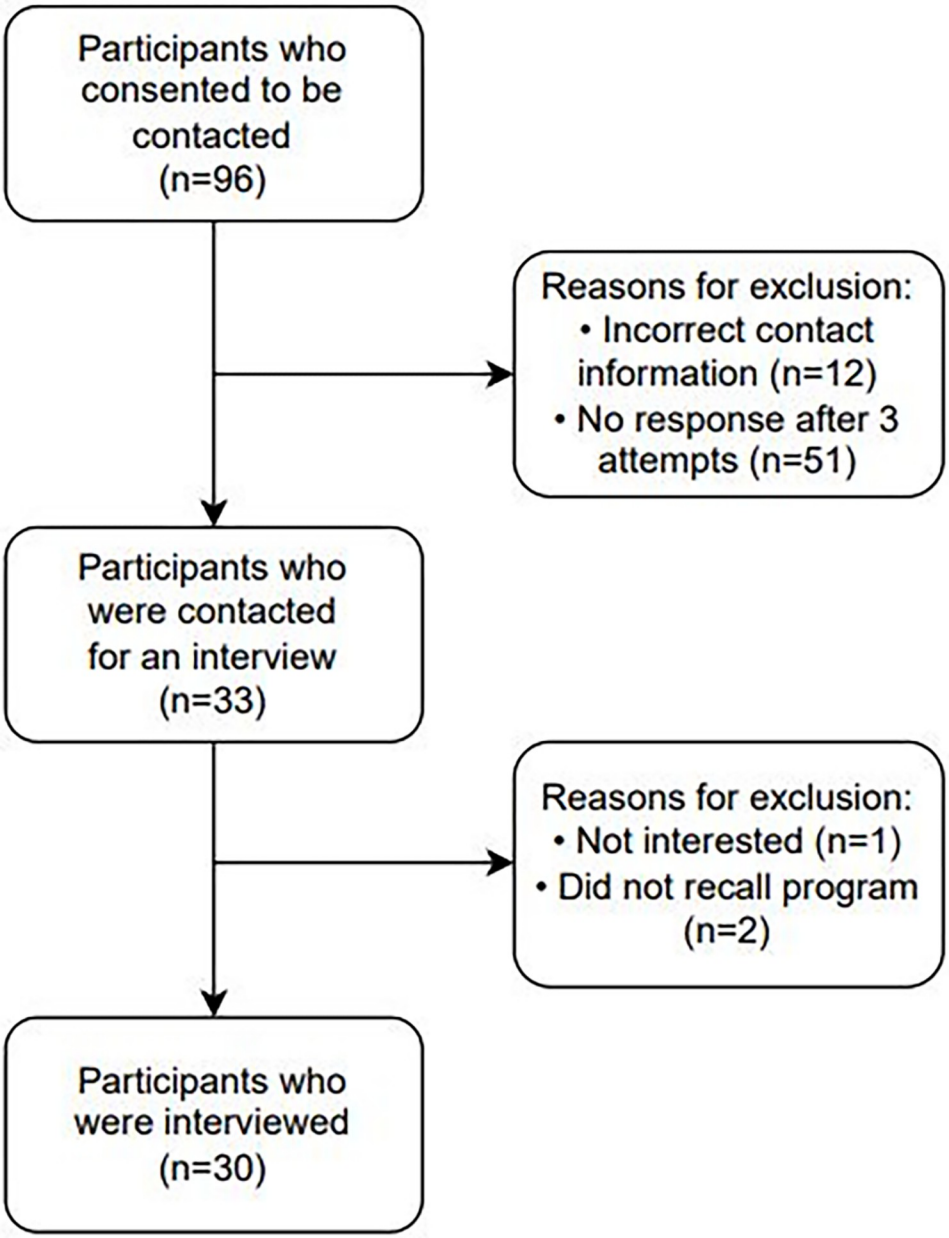
Study participant flow chart.

Ninety-six individuals consented to be contacted by the research team, 33 individuals were successfully contacted, and 30 completed an interview (see Fig 1 for study flowchart). Interviews ranged from 20 to 60 minutes.

Participant characteristics are outlined in Table 1. Of these 30 participants, 4 withdrew from the program for reasons such as an inability to do the exercises due to high pain levels (n = 1), the pace of the class (n = 2), and family illness (n = 1). Most participants had previous experience with non-surgical OA treatments such as physiotherapy, joint injections, medications, massage, chiropractic, or acupuncture. Prior to GLA:D^TM^, the majority (70%) reported they did not have prior experience with OA-specific programming. However, more than half of the participants (63%) maintained some level of physical activity through fitness classes such as yoga, aquafit, and television exercise program, and active outdoor lifestyle. Participants identified personal health issues and other circumstances such as family illness and accessing recreational facilities as factors that impacted their ability to keep active. Participants learned about the GLA:D^TM^ program from a variety of sources including health care clinics, health care providers, family and friends, and the media. Both internal (e.g., desire to stay active, desire to get better, interest in delaying or preventing surgery) and external factors (e.g., provider reputation, provider recommendation, positive previous experiences with exercise programs) were identified as motivating participants to consider participating in the GLA:D^TM^ program.

**Table 1:** Description of study and program participants (n = 30).

Participant and Program Descriptors	n (%)
**Sex**	
Female	21 (70)
Male	9 (30)
**Participant Location**	
Urban setting or metro setting	13 (43)
*(population of 25*,*000 or more)*	
Rural setting	17 (57)
*(population less than 25*,*000)*	
**Type of OA**	
Attended GLA:D for hip OA	8 (27)
Attended GLA:D for knee OA	22 (73)
**Physical Activity Participation**	
Participated in physical activity prior to GLA:D	19 (63)
Did not participate in physical activity prior to GLA:D	11 (37)
**GLA:D Payment Model**	
Attended a publicly paid program	24 (80)
Attended a privately paid program	6 (20)
**Program Attendance**	
Completed the program, but missed classes	7 (23)
Completed the program, no missed classes	18 (60)
Did not complete the program	5 (13)

### Participants’ description of the GLA:D^TM^ program

The GLA:D^TM^ program was described, positively and negatively, by participants in relation to the following program aspects: exercises, information and content, the group format, providers, and the venue. In general, participants had a positive experience of the GLA:D^TM^ program. The program was described as excellent, very good, good, and beneficial (see S2 File for a table containing quotes associated with each descriptor), for example, Participant 12 felt the program was excellent as demonstrated in the following quote:

*“I just think it’s an excellent program…being made aware of the things that you can do…I couldn’t say enough about it*, *that was kind of my experience*.”

*(Participant 12)*.

The enthusiasm for the program was also reflected in an interest to repeat the program and/or self-identifying as champions or advocates for the program, as exemplified by the quote from Participant 1 and Participant 17:

*“I loved it*. *I think everybody should do it…I’m a big promoter of the program*, *absolutely*. *Because I’m at a level right now where I’m thinking*, *I don’t need surgery*! *Seriously… [the provider] even asked me if I would speak at the next class*.”

*(Participant 1*)

*“I think it was offered again this year*. *And I actually looked into enrolling again*. *But they had a full complement*. *But I would have been happy to do it again*.”

*(Participant 17*)

#### Exercise

The exercise aspect of the program was viewed as progressive, modifiable, challenging and comprehensive; however, some noted that there was a need to be more accommodating to those with comorbidities. Participants came to the program with various strength, fitness, and ability levels. Hence, for some, the exercises were not challenging enough while others struggled with the pace or exercise levels, precluding their full participation in the program, as reflected in the experience of Participant 26:

*“And they got onto it a lot faster than me*. *So after I thought about it a while*, *I thought*, *…it’s not the*, *the pace*, *it’s that I’m not keeping up…that was another reason why I quit*. *I just thought I can’t do this anymore because…I was feeling like I was kind of*, *I don’t know*, *being a nuisance*. *They didn’t make me feel that way*, *I felt that by myself*.” *(Participant 26)*

Some participants enjoyed the focus on proper technique while others were impatient with this focus and wished to move more quickly through the exercises. Almost half of participants were less than satisfied with certain aspects of the program; they did not feel that GLA:D^TM^ offered anything new or unique. The same exercises had been previously provided by a physical therapist or were a component of other programs they had completed. Three participants reported sustaining an injury or increased levels of pain because of the GLA:D^TM^ exercise. While they were dissatisfied about not getting the results they wanted, they still spoke positively about the program itself, as exemplified in the quote from Participant 11,

*The exercises were really good*, *it’s just that I couldn’t do them*. *My left leg just could not do them*. *Because there were some where you had to stand on your leg and then do other exercises with the other leg*. *And I just*, *I could not do it*. *I just couldn’t*. (Participant 11)

Two of these three participants indicated that the program might not be a good fit as they had severe OA and highlighted that it might have been more successful for them if they had received the program when they had more moderate OA.

#### Information and content

Overall, the information and content of the program was perceived as useful as it either reviewed known information, confirmed existing knowledge, reiterated the benefits of exercise, or provided new and interesting information. Participants felt this information was helpful across many levels of knowledge of group participants. For some, however, the fact that the program did not offer new information or useful insights regarding OA and its management was not perceived as a beneficial aspect of the program and not a good use of time, as exemplified by Participant 27:

*“I didn’t think it was really necessary because none of us really know what’s going on with us*. *We want to—we want the cure to start*. *I didn’t think that we needed two… (education) sessions of it*, *myself*. *You know*, *I think one session to tell us what they plan on doing*. *And…how it might affect us*…*I think that would have been plenty*.*”*

(*Participant 27)*

#### Group format

While most participants viewed the group format as positive because it provided opportunities to share, compare, learn from, and motivate each other, for example Participant 4 found the group format to be supportive and relatable,

*“I liked the small group size*. *I liked the fact that the*, *the say*, *eight or nine other people in the group are people going through the same problems I’m going through*. *So it was a very supportive atmosphere*.*”* (Participant 4)

In contrast some participants perceived that the group format limited the time and attention from the provider and resulted in some unanswered questions. For example, Participant 26 found the group format to be limiting as exemplified in the following quote,

*“I did find it very helpful*. *But it seemed to me like because there was—I don’t know*, *there was probably eight or 10 or us there*. *Um a lot of times it seemed like everybody really didn’t get a chance to… ask their questions and have them answered…”* (Participant 26)

#### Providers

The providers (i.e., physical therapists, kinesiologists) were viewed in various ways by the participants. When viewed positively, providers were described as trustworthy, compassionate, and a good leader who helped them develop a plan and provide modifications. Participant 10 described how their provider was able to provide modifications to the program to better suit the different needs of the group,

*“[the provider] was excellent as far as presenting the program and helping us with the exercises*, *because people were all at different levels of mobility…there were some things I could do a lot better than some of the others*.*”* (Participant 10)

Participants also experienced providers who were not engaged during the lectures and at times inattentive during the program’s exercise portion or provided limited or ineffective demonstrations. For example, Participant 30 noted that their provider seemed disinterested in the program and that impact their interest,

*“I guess like they (GLA*:*D provider and assistant) didn’t seem very interested in it*. *And uh*, *transferred to me*. *I thought*, *if they’re not interested in it… why should I be interested*? *If I’d seen more interest*, *I might have felt that it was worth more*. *But I didn’t see any interest in me or*, *to be quite honest*, *the program*.*”* (Participant 30)

#### Venue

The venue descriptions varied substantially with some participants describing the space as lively, attractive, and suitable. For example, Participant 26 described their venue as well-equipped and beautiful,

*“…the venue is beautiful… where we exercised*, *it was*, *you know there was quite a few of us in there but it was…well equipped and everything*. (Participant 26)

While others described it as cramped, busy, and disrupted to others in the space, yet, this was not always viewed in a negative light, for example, Participant 15 found this busy atmosphere to be motivating but prevented group socializing as demonstrated in the following quote,

*“I can’t say there was all that much socializing going on since we were part of the fitness*, *the gym and there was lots of other stuff going on as well*. *So you felt like you were in a pretty lively atmosphere*. *And that was a plus of the program too… We certainly disrupted some of the other people’s routines…”* (Participant 15)

Access to equipment also varied by venue as some participants had access to helpful equipment such as stationary bikes while others had no additional equipment.

#### Emergent themes

**Fig 2:**
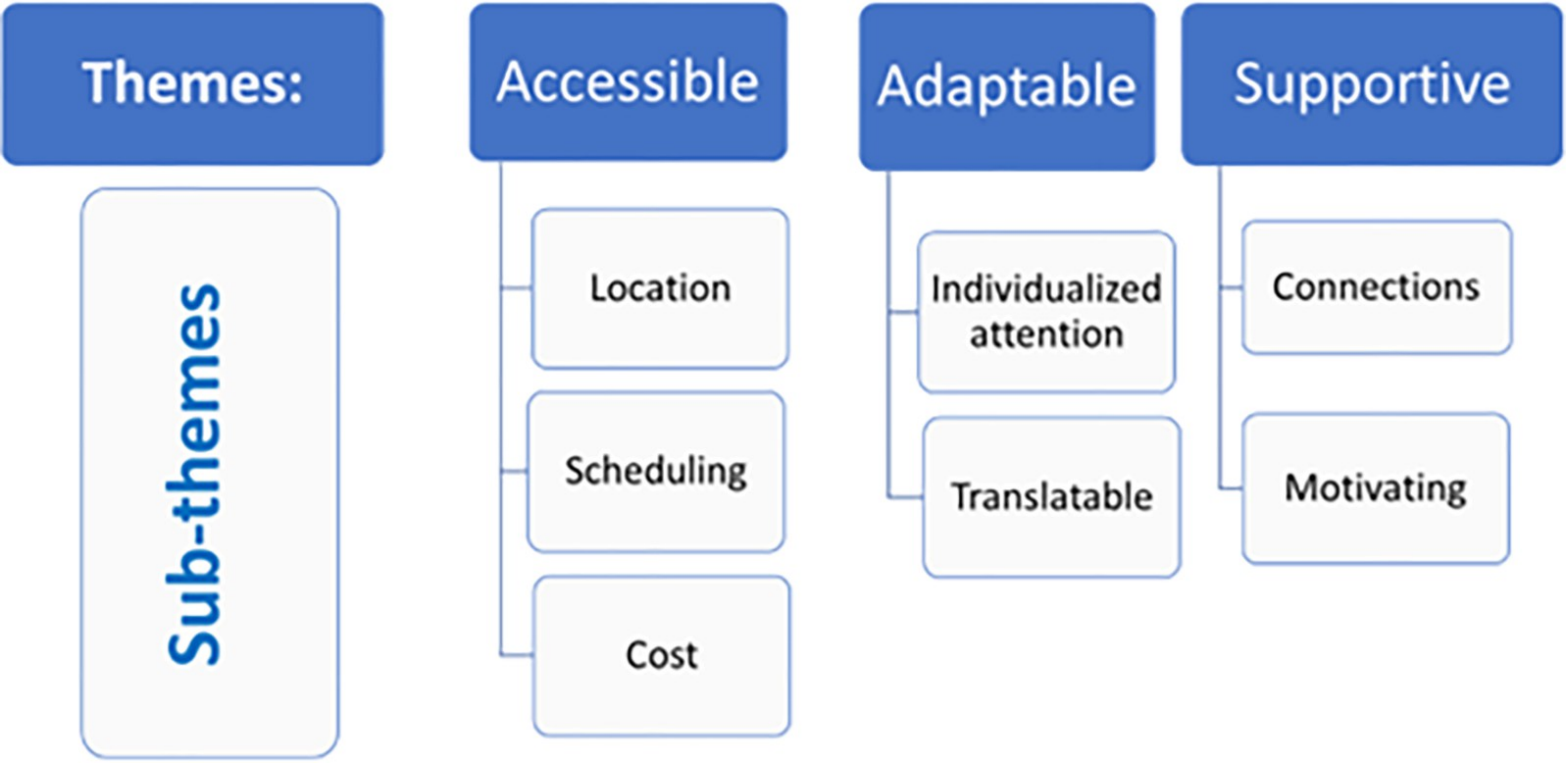
Emergent themes and sub-themes reflecting the experience of individuals who completed the GLA:D^TM^ program.

Participants’ ability to participate in the GLA:D^TM^ program and its utility in relation to their daily lives is reflected in the three emergent themes and related sub-themes: accessible, adaptable, and supportive (see Fig 2).

Selected supporting quotes for this section are presented in Table 2.

**Table 2:** Selected supporting quotes for emergent themes (accessible, adaptable and supportive) and sub-themes.

Theme	Sub-theme	Quote reference	Quote
Accessible	Location	1	“… And I know that the [clinic name] was less expensive. But there was going to be a lot more people in the class. And it was uh considerably farther for me to drive. And that was another consideration… So that was the reason for my decision.” (Participant 29)
		2	“…they started at the [town name] Health Centre, that’s where they did the evaluation. And then we continued … at what’s called the [town name] Rec Centre. They have a gym and a big space… [the provider] was able to connect with them…I don’t know how it would have worked right at the [town name] Health Centre. Because she wouldn’t have had the equipment there. The bicycles…a big bonus for the program.” (Participant 18)
	Scheduling	3	“…the time of the year was uh, really beneficial for me because if it had been snowing or icy or whatever, I’d have been really reluctant to go because falling once on the ice, and having to put up with the pain in my knee for a year, I just … very cautious. So the time of the year was really good.” (Participant 26)
	Cost	4	“…what kind of value to put on your physical well-being? Well, I still have to keep a roof over my head. And, and gas in the car, do you know what I mean? So you have to find the balance.” (Participant 1)
		5	“…they didn’t seem to have any interest in me or the program…I thought it was kind of a research thing and I thought if it’s a research thing that I was paying a lot of money for basically to participate in a research thing where they weren’t doing much research. I mean they weren’t even watching us very much in our exercises and stuff, to track our progress. … I don’t know what you pay for physiotherapists, it’s probably cheap for physiotherapist. But I didn’t think that I got, like maybe $50 worth of anything out of the program.” (Participant 30)
		6	“I would have never done it…it’s a lot of money, like that’s almost a whole year of a gym membership…you can get um, quite a bit of help at the gym or you can pay for personal trainer for less than that amount. …when I pay for a chiropractor treatment, a physio treatment, a massage therapy treatment, these people are working one on one with me. I’m not having to share them with even one other person. So if it wasn’t covered it’s not the type of program I would be interested in.” (Participant 32)
		7	“…initially I thought it was pricey. And then I thought about how much I’d paid for all my other physiotherapy sessions, and thought well, you know, what? This actually isn’t that pricey. I was a little concerned doing it with other people because I wanted to make sure a.) I was going to get my money’s worth. And b.) it was going to be um beneficial…now all I can say is it was a deal.” (Participant 29)
Adaptable	Individualized	8	“She (the provider) was very helpful, … very uh watchful that I didn’t overdo something…she let me work just to the point where I could, or let me do what I thought I could. And you know, there was no pressure there, no.” (Participant 18)
		9	“These little exercises are fantastic! And they’re not that easy. They’re simple, but they are not that easy. And if you do them right, they can have a lot of benefits. But, but without the help to do them right, I would think that most people would do them wrong.” (Participant 20)
		10	“…I didn’t have real, real serious pain all of the time…so it was a bit easier for me to do the exercises right out of the gate…[the provider] made sure that I had harder modifications, you know, fairly early on, so um I was able to, to do a little bit more, I guess.” (Participant 5)
		11	“But she was very careful to notice which areas we seemed, you know, to be struggling with or needed the most help with, let’s put it that way. She gave each of us individually a little extra attention in those areas, which was, which was terrific. That’s what I liked about her.” (Participant 28)
	Translatable	12	“The other thing I really liked about the program was you could cater it to do it at home. It was not difficult to set up and them here at home, which is what I did”. (Participant 12)
		13	“…the worksheet that the provider gave me was nice because what I did is I just took that worksheet that we did on our Tuesdays and Thursdays and I split it in half so that I could do Monday half of it, Tuesday the other half, Wednesday the first half, Thursday—you know… So it’s not like I’m just doing half an exercise. I’m just trying to fit all the exercise I need in a day.” (Participant 32)
Supportive	Connection	14	“…the fact that the exercises are specifically designed and you’re doing them in a group. I’ve gone through physio and doing it alone and I really liked the small group atmosphere better. And I think that would probably encourage you to continue and to go back”. (Participant 4)
		15	“And it’s nice to have a group of people that are dealing with the same thing, because going to a gym or going anywhere else, they don’t… have arthritis don’t understand what it’s like.” (Participant 24)
		16	“…it’s kind of frustrating to have a really cute, nice girl tell us, well, if you just exercise more you won’t have pain.” (Participant 27)
	Motivation	17	“It’s so much easier to exercise when you’re with other people doing the same thing. And you can See other people improving and so it’s very motivational” (Participant 11)
		18	“…it was quite incredible, actually. I was amazed…I did the best of the whole group and there was a real jock. Like she was really athletic. I did better than her coming out of that—not that we were competing. But I did more because I’d actually improved that much. And I was amazed. I know.” (Participant 31)
		19	“…they found me ways of doing the same thing other ways, which I thought was pretty amazing. … they were very encouraging without pushing.” (Participant 24)
		20	“I’d say is the [providers] that we worked with were great. They let you move at the rate that they thought was best for you. And they kind of pushed me a little bit to go further, and told me what I could do it. And once I reached a certain level, and if things were going good, they just upped it there”. (Participant 21)
		21	“…it pushed me to do more than what I would have. I could easily have been lazy and just kind of … gotten to an easy—a comfortable point. Whereas um this one pushed me. And uh I have to admit, I needed that. It was great.” (Participant 29)
		22	“I guess like they didn’t seem very interested in it. And uh, transferred to me. I thought, if they’re not interested in it, really, and I don’t see anything really happening here… why should I be interested?” (Participant 20)

#### Accessible

The delivery of the program was perceived as accessible by nearly all participants, and was discussed in relation to clinic location, scheduling, and program cost. Travel distance was a key factor in the decision to enroll in the program. The physical location of the program, whether a clinic, health centre, or community recreational facility or gym, was agreeably situated for most participants who were within walking distance, had access to a transportation service, or who drove. For some participants in rural settings, the required travel distance to attend the program was a challenge; however, it did not preclude program participation. Having the program at multiple locations in larger centers enhanced access as it provided participants with choice to accommodate their preferences (see Table 2, quote 1).

Facility features such as ample exercise space and equipment (for example, stationary exercise bikes that enable the recommended warm-up), was another key consideration (see Table 2, quote 2). Scheduling of sessions was also another consideration for most participants. The program was offered on days or at times that were convenient. Consistent class schedules–the set day and time–was perceived as advantageous as it kept participants on track and accountable. Two classes a week was perceived as the right frequency as it kept people engaged while providing enough time between classes to enable appropriate recovery. Scheduling in relation to time of year was also an important consideration for some as seasonal variations had a direct impact on their ability to participate. For example, scheduling during the winter months would preclude their participation outside of their home due to concerns about falling on ice (see Table 2, quote 3).

Cost was not a significant consideration as most participants did not pay for the program out-of-pocket; fees were covered by public health insurance (n = 23) or private health insurance (n = 2). Although fees did not appear to be a barrier to accessing the program for this participant sample, some recognized that in situations of low or fixed incomes, cost would impede the ability or decision/choice to do the program (see Table 2, quote 4).

When hypothetically asked what they would pay for the program, four individuals (two of whom attended a public clinic and two who paid for the program at a private clinic) indicated they would not pay for the program. Reasons included poor experience and too expensive (see Table 2, quotes 5, 6). After completing the program, 25 participants, 19 of whom completed the program in a publicly funded setting, expressed they would pay for the program, with acceptable fees ranging from $50 to $400. The monetary value of the program, including those who would not pay for it, was informed by factors such length of the sessions, perceived effectiveness, and comparisons to other services (physiotherapist treatments, gym membership, fitness trainer, group fitness classes) used to manage OA symptoms (see Table 2, quote 7).

#### Adaptable

The program was adaptable to individuals’ needs, reflected in two sub-themes: individualized and translatable. The exercises could be individualized and thus modified to accommodate various activity levels and individual participants’ needs, enabling broad participation irrespective of ability level. Participants could work within their ability levels without pressure, enabling them to develop strength and gain confidence (see Table 2, quotes 8 and 9).

Many participants recognized the provider’s ability to simultaneously progress the group and address individuals’ specific needs. Although the program is not intended to be one-on-one with the provider, individual participants were monitored and provided with instruction, feedback, and appropriate modifications for their ability level (or limitations). In addition, the group size (often 6 to 10 participants) was perceived to enable the time and attention needed to address the individual’s needs. Yet, some participants felt the group format decreased the amount of time spent with each individual and some would have liked additional assistance and demonstration of the exercises. The individualized attention within a group context was perceived as a key program benefit (see Table 2, quotes 9,10,11).

Participants felt the exercises were translatable and were able to apply the exercises in other activity settings or contexts. They reported continuing the exercises outside of the structured program, albeit the frequency and consistency was variable. In addition, participants appeared to selectively choose the exercises they continued after completing the program. Some also selectively incorporated the GLA:D^TM^ exercises into existing exercise routines at the pool, gym, or home setting (see Table 2, quotes 12 and 13).

#### Supportive

The following emergent sub-themes underpin this aspect: connections and motivation. The group format enabled new relationships and a sense of comradery among participants. The sense of connection was based on a shared experience of living with OA and participating in the program. The shared experience of living with OA provided a common ground through which participants had a unique understanding of each other and were able to engage with one another (see Table 2, quote 15).

The program format offered an opportunity to learn from others, and further engage with, and absorb the program content (see Table 2, quote 14). Exercising and learning with others in a similar situation also gave the program credibility. It was important for providers to manage participants’ perception of them, as it impacted how participants perceived the program. For example, learning exercises from a young(er) provider not struggling with OA made some approach the program with some skepticism, impacting their level of engagement in or uptake of the program (see Table 2, quote 16).

For almost all, participating in GLA:D^TM^ was described as a motivating experience, influenced by other participants and providers. Within the group setting, the presence of others created a reference point, which challenged participants and generated a form of healthy competition for some. Watching others struggle created a realization for many that they were not alone in their personal physical struggles and perceived challenges were not insurmountable. Observing progress and improvements of others was identified as a strong motivating factor by many participants (see Table 2, quotes 17 and 18). Providers created a supportive environment where participants felt enabled to work at their own pace and did not feel pushed (see Table 2, quote 19) or pressured to go beyond their comfort level. Providers also played an important role in creating an appropriately challenging yet encouraging environment, which motivated participants to try new things and do their best (see Table 2, quotes 20 and 21). Active engagement and expressed interest of providers in the program and with the participants was frequently described as important. When these aspects were lacking, motivation to do the program was directly impacted (see Table 2, quote 22).

### Recommendations for improvement

Areas of possible improvement related to screening assessment, session organization, follow up classes, and earlier intervention, and are shown in Table 3.

**Table 3:** Emergent recommendations for improving the GLA:D^TM^ program.

Sub-Theme	Key Points	Supporting Quotes
Screening assessment	Better ability to identify individuals not suitable for the program (e.g., pain levels too high; frail; significant limitations in mobility) Better management of expectations of participants knowing which would have minimal benefit from the program	*“…the program as it exists right now…was not a great benefit to me*. *…they need to do a better job of assessing the patient before the patient starts the program*, *to find out if their arthritis indeed has maybe gone too far*. *(Participant 28)”*
Session organization	Exercise sessions taught in clusters to better support the learning process. For example, do 2–3 sessions and provide a week or two where participants practice. Education sessions can be shortened and condensed; spend more time on the exercises.	*“They could run it and like do a few…* *sessions*, *and then give people a couple weeks to* *work on it*, *and then come back*. *So there’s more* *of a long-term thing*.*” (Participant 20)* *“I found it useful*. *… I think it could* *have been a one-day education*. *And exercises to* *me were more important uh than sitting there*, *looking at pictures of bones*. *Nice to understand*, *but in my case*, *it wasn’t vital*.*” (Participant 24)*
Post-program follow-up session(s)	Offer refresher classes after program completion. May be optional and provide an opportunity to ensure exercises are being done correctly, recall, and learn more advanced exercises.	*“So hopefully at some point they*, *they will have a* *refresher course*. *Because I think that’s more* *important than anything*, *because why spend all* *that money teaching all these people how to do* *these things*, *and now have a follow up to* *make sure they’re on board*, *or where they’re* *having problems with or whatever …”* *(Participant 20)*
Use proactively	Introduce the program at earlier stages of OA, before symptoms become severe or significantly limiting, for better overall management of the disease	*“I think that the GLAD program is good for* *people who…have moderate levels of osteo-arthritis*. *But… if it’s severe*, *um I think it’s just*.* *.* *. *it’s too far gone*. *And I was too far gone*.* *.* *.*” (Participant 11)* *“I see this program as a good program especially* *earlier on in uh arthritic conditions… I would somehow try to get to people who*, *well even for* *people who don’t have it*, *but are having some* *joint issues*, *strength and so on*, *…I think they would very well be able* *to keep uh full-blown arthritis at*, *at bay*.*”* *(Participant 16)* *“I do hope more people can benefit from it*. *And* *that people will take it before they get to the point* *where there’s no return…*.*”* *(Participant 17)*

## Discussion

This qualitative study explored the perceptions and experiences of individuals living with OA who participated in the GLA:D^TM^ program for knee and hip OA. Study participants were satisfied with the GLA:D^TM^ program and felt that it was appropriate for management of their OA symptoms. Accessibility, adaptability, and supportiveness emerged as important themes that reflected the participants’ experience. Recommendations for improving participant experience centered on better screening to ensure the right individuals take the program, rearranging session organization to maximize continuity, offering post-program follow-up, and ultimately using GLA:D^TM^ as a proactive intervention for conservative OA management (see Table 3).

Similar studies have reported high satisfaction with the GLA:D^TM^ program for hip and knee OA [14, 31]. However, our findings provide insights as to why the program was satisfactory, why, in some cases, it was not, and how to potentially improve the program from the patient’s perspective. A group format has been reported to be a facilitator for physical activity among people living with OA, particularly when individuals are exercising [32]. Participants consistently reported that the group context added to the positive program experience. The group format also provided a sense of connection and motivation to achieve something that most found challenging. Our participant findings align with findings from previous evaluations of the GLA:D^TM^ program for hip and knee, where the in-person group format was identified as motivating by both the participants [19] and the providers [20].

While the group format was a clear benefit, at times it created some difficulties as providers aimed to balance individual-level attention while progressing the group. Providers of the GLA:D^TM^ program reported that class size was a barrier to program delivery as they often needed more time with participants at the beginning to explain the exercises satisfactorily for participants, provide more intense supervision, and address concerns and questions [14]. Our participants recommended improved screening assessments to better identify individuals who may benefit from the GLA:D^TM^ program. The GLA:D^TM^ program may not meet the needs of all participants even if they fit the medical inclusion criteria, as the group format delivery might not be sufficiently individualized for participants with severe pain and advanced stages of OA. In those limited cases, participants may benefit from more individualized programs. While this area of research is still developing, a recent randomized clinical trial that compared group vs. individualized program outcomes, among veterans with knee and hip OA, found no evidence of a difference between the two models [33]. Therefore, adaptations to the screening assessment for the GLA:D^TM^ program may improve appropriateness of selection for participants taking the GLA:D^TM^ program, and also assist in better managing the participant expectations, thus improving program outcomes and satisfaction. A more effective strategy to help individuals better manage their OA symptoms may be an earlier intervention with the GLA:D^TM^ program, prior to symptoms becoming severe [10].

Patient knowledge about OA is recognized and accepted as a critical factor to effective OA management [7, 34]. Further, lack of patient knowledge about OA and treatment options is often cited as a barrier to accessing appropriate treatments [19]. Participants of our study were knowledgeable about OA and the outcomes of their disease progression. The majority indicated they were already knowledgeable or at least familiar with the contents of the educational component of the program. Although the repetition of information was perceived positively by most, it was not perceived as new information; the information was perceived as new by only a few participants. Yet, the educational component of the GLA:D^TM^ program is critical as participants may perceive they have good knowledge of OA but use outdated and inaccurate terms to describe their condition. This was observed by Teo et al. [35] where participants of the GLA:D^TM^ program believed they had adequate education and knowledge about OA but described their condition using terms such "wear and tear" and “bone on bone”. Further, in our participant group, experience with OA exercise therapy (structured or unstructured) was limited and highly variable. For almost all participants, GLA:D^TM^ was their first OA-specific exercise program. Thus, our findings suggest that an important consideration for OA education programming or campaigns is that awareness or knowledge may not be enough; knowing something and doing something is not always directly linked. This disconnect has been observed in a study examining the GLA:D^TM^ for Low Back participant experience where some participants understood the concepts discussed during the education component and understood how the exercises are done but struggled to integrate these learnings into habits [36]. Effective educational interventions may also need to provide opportunities for information to translate into action, meaningful uptake, and beneficial behaviour change. GLA:D^TM^ as a program that combines education and exercise, may have done this effectively in that the knowledge is experienced and lived by the participants through the exercise session, which likely leaves a much more engrained and lasting impression of that knowledge. Therefore, re-organizing sessions to shorten the educational components while providing more time for practicing the exercises with supervision may better suit the educational needs of the GLA:D^TM^ participants. To this end, refresher sessions may be a valuable aspect of the education provided through GLA:D^TM^, offered to enable participants to transition the exercises to other environments and return for additional feedback or reminders after the program is completed.

Barriers to accessing health services are a key concern for health care administrators, health care professionals, and patients. Previous research identified the following as barriers to accessing the GLA:D^TM^ program: cost [11, 13, 19, 35], location, transportation, and parking [19]. Although some of these aspects were also acknowledged and discussed by our participants, these did not emerge as barriers in our participant sample but instead as access facilitators. The GLA:D^TM^ program in Alberta was implemented widely across the province; in most of the larger cities it was offered at multiple locations and in both private and public clinics, which provided participants with options. Clinic locations were also such that participants felt they could easily access the program by foot, bus, or car. Our study participants did not report any negative experiences around the program cost, possibly due to most participants having program access through public health care clinics (at no participant cost). Further, those who did pay out of pocket were reimbursed by private health care insurance. We do not know how many interested participants chose to not participate in the GLA:D^TM^ program due to concerns about or limitations due to out of pocket costs. However, our results suggest that providing access to the GLA:D^TM^ program in both private and public clinics enables choice and enhances access to the program, irrespective of socioeconomic status.

### Strengths and limitations

Quantitative evaluation of the implementation [11, 13, 14] and outcomes [12–15] of GLA:D^TM^ program is expanding globally, and while there is an emerging body of literature examining qualitative outcomes from a provider perspective [14, 20], there is a paucity of research focused on how the participant perceives and experiences the GLA:D^TM^ program [19, 20]. To our knowledge this is the first study to focus on the acceptability of the GLA:D^TM^ program from the patient’s perspective in Canada. These findings can help inform the population wide implementation of this program in other regions with similar contexts (i.e., public and private providers). Our study makes an important contribution to the literature on the GLA:D^TM^ program, specifically regarding its acceptability from the OA participants’ perspective.

This paper is the first to report the participants’ perspective regarding the group format delivery of the GLA:D^TM^ program for hip and knee. The present study has several strengths that are worth noting, providing new insights in relation to the implementation of the GLA:D^TM^ program. First, we included a broad sample of participants with few exclusion criteria to allow exploration of a diversity of experiences across demographic groups. Second, our sample included participants who received the GLA:D^TM^ program through both public and private clinics, which provided an opportunity to explore experiences across these two settings. Third, we examined participant experiences across a provincial implementation of the GLA:D^TM^ program rather than a single site. This enables transferability to other jurisdictions implementing this program. Finally, the present study is the first to examine acceptability of the GLA:D^TM^ program among participants and the first to examine participant experience qualitatively in Canada.

The present study also has several limitations to consider when interpreting the findings. First, we cannot fully present barriers or factors precluding program participation, as we did not collect data on those referred for the program but who did not register. Second, these findings may not apply to contexts where cultural beliefs differ from the Canadian context given that individual experiences with pain management, health behaviours, exercising in group settings, and disability vary across cultures [37, 38]. Third, interviews occurred from 1 month to 12 months after program completion. Although this timeline aligned with quantitative data collection, it may result in potential recall bias. We monitored for this possibility and two potential participants were excluded from the study due to their inability to recall the program. Fourth, given the various in GLA:D^TM^ program delivery across Canada, where healthcare is provincial jurisdiction, these findings may not be transferable to other provincial settings where implementation of the GLA:D^TM^ program may vary. Finally, we did not include people living with OA or GLA:D^TM^ program participants in the design of this research study or design of the interview guide; however, our overall evaluation was informed by patient advisors engaged with the Bone and Joint Health Strategic Clinical Network. Future GLA:D^TM^ evaluations should include representatives of the study population (e.g., GLA:D^TM^ participants) to maximize integrated knowledge translation and exchange.

### Conclusion

The GLA:D^TM^ program was received in a similar manner by participants in both the public and private health care setting. The program’s structure and content were perceived by participants as accessible, useful, supportive, and motivating. The group format was perceived as a program benefit for most, but not all individuals living with OA felt that it met their needs. Based on participants’ experiences, the GLA:D^TM^ program can be further improved by better screening for appropriate participants, providing access to the program earlier in the disease progression trajectory, ensuring educational content is responsive to participants’ needs, and introducing refresher sessions after program completion support long-term behaviour change.

## Supporting information

S1 Table Completed COREQ (COnsolidated criteria for REporting Qualitative research) [24] checklist. (DOCX)

S1 File GLA:D^TM^ patient semi-structured interview guide. (DOCX)

S2 File Selected participant quotes on positive experiences of the GLA:D program. (DOCX)
