# Supplementary material for: ‘I do hope more people can benefit from it.’: The qualitative experience of individuals living with osteoarthritis who participated in the GLA:D™ program in Alberta, Canada
Source: PLoS One. 2024 Feb 21;19(2):e0298618. doi: 10.1371/journal.pone.0298618 (PMC10881017; doi:10.1371/journal.pone.0298618)
Supplement: S1 Table — (DOCX) [file pone.0298618.s001.docx]

**S1 Table. Completed COREQ (COnsolidated criteria for REporting Qualitative research) Checklist**

| **Topic** | **Guide Questions/Description** | **Completion reference** |
| --- | --- | --- |
| **Domain 1**: Research team and reﬂexivity | | |
| *Personal Characteristics* | | |
| 1. Interviewer/facilitator | Which author/s conducted the interview or focus group? | Methods, Research team and reflexivity (page 7, para 1) |
| 2. Credentials | What were the researcher’s credentials? E.g. PhD, MD | Methods, Research team and reflexivity (pages 7-8, para 1) |
| 3. Occupation | What was their occupation at the time of the study? | Methods, Research team and reflexivity (pages 7-8, para 1) |
| 4. Gender | Was the researcher male or female? | Methods, Research team and reflexivity (page 8, para 1) |
| 5. Experience and training | What experience or training did the researcher have? | Methods, Research team and reflexivity (pages 7-8, para 1) |
| *Relationship with participants* | |  |
| 6. Relationship established | Was a relationship established prior to study commencement? | Methods, Research team and reflexivity (page 8, para 1) |
| 7. Participant knowledge of the interviewer | What did the participants know about the researcher? e.g. personal goals, reasons for doing the research | Methods, Research team and reflexivity (page 8, para 1) |
| 8. Interviewer characteristics | What characteristics were reported about the interviewer/facilitator? e.g. Bias, assumptions, reasons and interests in the research topic | Methods, Research team and reflexivity (page 8, para 1) |
| **Domain 2: S**tudy design | | |
| *Theoretical framework* | | |
| 9. Methodological orientation and theory | What methodological orientation was stated to underpin the study? e.g. grounded theory, discourse analysis, ethnography, phenomenology, content analysis | Methods, Study design (page 5, para 1) |
| *Participant selection* | | |
| 10. Sampling | How were participants selected? e.g. purposive, convenience, consecutive, snowball | Methods, Participant selection (page 5, para 1) |
| 11. Method of approach | How were participants approached? e.g. face-to-face, telephone, mail, email | Methods, Participant selection (page 5, para 2) |
| 12. Sample size | How many participants were in the study? | Methods, Data collection (page 6, para 1) |
| 13. Non-participation | How many people refused to participate or dropped out? Reasons? | Results, Participant description (page 8, para 1) |
| *Setting* | | |
| 14. Setting of data collection | Where was the data collected? e.g. home, clinic, workplace | Methods, Data collection (page 6, para 1) |
| 15. Presence of non-participants | Was anyone else present besides the participants and researchers? | Methods, Data collection (page 6, para 1) |
| 16. Description of sample | What are the important characteristics of the sample? e.g. demographic data, date | Results, Participant description (page 8, para 1) |
| *Data collection* |  |  |
| 17. Interview guide | Were questions, prompts, guides provided by the authors? Was it pilot tested? | Methods, Data collection (page 6, para 1) |
| 18. Repeat interviews | Were repeat interviews carried out? If yes, how many? | N/A |
| 19. Audio/visual recording | Did the research use audio or visual recording to collect the data? | Methods, Data collection (page 6, para 1) |
| 20. Field notes | Were ﬁeld notes made during and/or after the interview or focus group? | Yes, field notes were taken post-interview and during analysis. |
| 21. Duration | What was the duration of the interviews or focus group | Methods, Data collection (page 6, para 1) |
| 22. Data saturation | Was data saturation discussed? | Methods, Data collection (page 7, para 1) |
| 23. Transcripts returned | Were transcripts returned to participants for comment and/or correction? | Methods, Data analysis (page 7, para 1) |
| **Domain 3**: Analysis and ﬁndings | | |
| *Data analysis* | | |
| 24. Number of data coders | How many data coders coded the data? | Methods, Data analysis (page 7, para 1) |
| 25. Description of the coding tree | Did authors provide a description of the coding tree? | Figure 1, Results (page 4) |
| 26. Derivation of themes | Were themes identiﬁed in advance or derived from the data? | Methods, Data analysis (page 7, para 1) |
| 27. Software | What software, if applicable, was used to manage the data? | Methods, Data analysis (page 7, para 1) |
| 28. Participant checking | Did participants provide feedback on the ﬁndings? | Methods, Data analysis (page 7, para 1) |
| *Reporting* | | |
| 29. Quotations presented | Were participant quotations presented to illustrate the themes / ﬁndings? Was each quotation identiﬁed? e.g. participant number | Results (pages 11-27) and Tables 3 and 4 |
| 30. Data and ﬁndings consistent | Was there consistency between the data presented and the ﬁndings? | Supporting quotes are embedded in the text in the results section and presented in Table 4 |
| 31. Clarity of major themes | Were major themes clearly presented in the ﬁndings? | Table 3 (pages 17-20) |
| 32. Clarity of minor themes | Is there a description of diverse cases or discussion of minor themes? | Positive and negative quotes associated with aspects are presented in Table 2 (10-11). Sub-themes are presented in Table 3 (pages 17-20) |
