# Supplementary material for: ‘I do hope more people can benefit from it.’: The qualitative experience of individuals living with osteoarthritis who participated in the GLA:D™ program in Alberta, Canada
Source: PLoS One. 2024 Feb 21;19(2):e0298618. doi: 10.1371/journal.pone.0298618 (PMC10881017; doi:10.1371/journal.pone.0298618)
Supplement: S1 File — (DOCX) [file pone.0298618.s002.docx]

**S2 File. GLA:D Patient Semi-Structured Interview Guide**

Why did you sign up to take GLA:D?

What factors contributed to your decision to sign up to GLA:D?

*How did you come across the GLA:D program?

Have you done any other exercise programs for your OA?

What were they?

Where they similar to or different from GLA:D? How?

What was your experience of the GLA:D program? The education session? The exercise?

Did you find the exercise/education useful?

Do you think you benefited from doing GLA:D? how?

Would you do it again?

*Have you maintained the exercises after the GLA:D program?

What did you think of the cost of GLA:D?

*How much would you be willing to pay for GLA:D?

What other factors are important to the idea of “willingness to pay”

What would make it easier for you to take a program like GLA:D?

** Indicates questions added to the original semi-structured interview guide*
