# Supplementary material for: ‘I do hope more people can benefit from it.’: The qualitative experience of individuals living with osteoarthritis who participated in the GLA:D™ program in Alberta, Canada
Source: PLoS One. 2024 Feb 21;19(2):e0298618. doi: 10.1371/journal.pone.0298618 (PMC10881017; doi:10.1371/journal.pone.0298618)
Supplement: S2 File — (DOCX) [file pone.0298618.s003.docx]

**S3 File. Selected Participant Quotes on Positive Experiences of the GLA:D Program**

| **Descriptor** | **Quote** |
| --- | --- |
| Excellent | “…the program itself was excellent. Is excellent. Yeah. And, and I mean, we’re so lucky to have a program like that in the [location name].” (Participant 11)  “… just think it’s an excellent program. And, you know, really if it keeps people out of the, out of surgery, I would think that’s a real benefit….and being made aware of the things that you can do. And uh I just thought it was—I couldn’t say enough about it, that was kind of my experience.” (Participant 12)  “I think it was a great program. I can’t say anything negative about it. I really can’t.” (Participant 1) |
| Very Good | “I thought it [the GLAD program] was very good. I thought it was excellent…I’ve often told people it was good.” (Participant 14) |
| Good | “I see this program as a good program, especially earlier on in uh arthritic conditions…when there’s initial twinges…” (Participant 16) |
| Beneficial | “It [the GLA:D program] was very beneficial. This is one of a few proactive measures that our Health Care system is taking to keep people out of the system instead of being reactive and just treating illnesses and conditions with surgeries and drugs. So this is something I’ve been waiting for, for a longtime, and we need much, much more of such programming available. So I’m very happy with it.” (Participant 18)  “I think that it’s a very, very beneficial program. Especially for those of us who are aging and have had different pains and…limited our physical abilities or physical activities. I think they would very much be of benefit.” (Participant 2)  “Well it [the GLAD program] actually took away some of the pain because I was doing things wrong. So that helped. And as far as strengthening, I was able to strengthen the muscle properly as opposed to just causing more pain and stress to it. So I just thought that was really very important and beneficial.” (Participant 29) |
